# Muon dynamic radiography of density changes induced by hydrothermal activity at the La Soufrière of Guadeloupe volcano

**DOI:** 10.1038/srep33406

**Published:** 2016-09-15

**Authors:** Kevin Jourde, Dominique Gibert, Jacques Marteau, Jean de Bremond d’Ars, Jean-Christophe Komorowski

**Affiliations:** 1 Institut de Physique du Globe de Paris (CNRS UMR 7154), Sorbonne Paris Cité, Paris, France; 2 Institut de Physique Nucléaire de Lyon, Univ Claude Bernard (UMR 5822 CNRS), Lyon, France; 3 OSUR - Géosciences Rennes (CNRS UMR 6118), Université Rennes 1, Rennes, France; 4 National Volcano Observatories Service, Institut de Physique du Globe de Paris (CNRS UMR 7154), Paris, France

## Abstract

Imaging geological structures through cosmic muon radiography is a newly developed technique which shows a great potential in volcanology. Here we demonstrate that muon radiography permits to detect and characterize mass movements in shallow hydrothermal systems of low-energy active volcanoes like the La Soufrière lava dome. We present an experiment conducted on this volcano during the Summer 2014 and bring evidence that very important density changes occurred in three domains of the lava dome. Depending on their position and on the medium porosity the volumes of these domains vary from 1 × 10^6^ m^3^ to 7 × 10^6^ m^3^. However, the total mass budget remains approximately constant : two domains show a mass loss (Δ*m*∈ [−0.8;−0.4] × 10^9^ kg) and the third one a mass gain (Δ*m*∈ [1.5; 2.5] × 10^9^ kg). We attribute the negative mass changes to the formation of steam in shallow hydrothermal reservoir previously partly filled with liquid water. This coincides with the emergence of new fumaroles on top of the volcano. The positive mass change is synchronized with the negative mass changes indicating that liquid water probably flowed from the two reservoirs invaded by steam toward the third reservoir.

The La Soufrière of Guadeloupe volcano belongs to the Lesser Antilles volcanic arc formed by the subduction of the North American Plate beneath the Caribbean Plate. La Soufrière is the last lava dome in a series of dome extrusions and collapses^1^,^2^,^3^. During the last 8500 years, between 2 and 5 of the 8 collapses that occurred at La Soufrière of Guadeloupe have generated laterally-directed explosions caused by depressurisation (volcanic blasts) of magma and hydrothermal fluids that spread laterally at high speeds (up to 100–235 m.s.^−1^) over the volcano flanks. These events caused partial collapses and triggered small laterally-directed hydrothermal explosions associated with significant exurgence of hot acid pressurized hydrothermal fluids contained in superficial reservoirs located inside the lava dome^1^,^4^.

The last eruption occurred in 1976–1977 and is considered as a failed magmatic event : a small andesitic magma volume stopped its ascent about 3 km beneath the surface^5^,^6^. Following this event, degassing, thermal flux and seismicity progressively decreased down to their lowest levels by 1990. By the end of 1992, a resumption of the fumarolic activity at the summit of the dome, of the shallow seismicity, and of the temperature of thermal springs around the dome was observed. In 1998, the sudden onset of high-flux chlorine degassing from the Cratère Sud vent constituted a conspicuous change in the magmatic-hydrothermal system behaviour. In 1997, a small boiling pond formed at the Cratère Sud with extremely acid fluids (minimal pH ≈−0.8) corresponding to the azeotrope point of hydrochloric acid. A similar larger boiling lake was discovered in 2001 in the Tarissan pit. In 2003, the acid pond at the Cratère Sud was replaced by a strongly degassing vent while Tarissan acid pond continues to exist until now. Since then, a significant increase of the dome fumarolic activity was observed^7^,^8^,^9^. Indeed a new active region appeared to the North-East of the Tarissan pit during the 2014 Summer^8^, the North-Napoleon fumarole, and two old pits, the Gouffre Breislack and the Gouffre 56, have seen their activity rising^9^. All these sites are reported on Fig. 1.

The spatio-temporal evolution of active areas located onto the lava dome may be caused by the progressive sealing of previously active flow paths. This sealing results from the combined effects of hydrothermal activity and heavy rains (≈6–7m.y^−1^) that favour fluid mineralization by magmatic gas and the formation of clayey material that progressively fills open fractures. Sealing causes fluid confinement and over-pressurization leading to the opening of new flow paths. The observed increased flux of chlorine-rich vents at the summit and the episodic chlorine spikes recorded in the Carbet and Galion hot springs^6^ can be due to the sporadic injection of acid chlorine-rich fluids and heat from the magma reservoir or magma intrusions at depth into the hydrothermal reservoirs^6^.

Purely hydrothermal blasts are as mobile as their magmatic counterparts. Small-scale laterally-directed explosions could have caused many fatalities at Tongariro (New-Zealand) in 2012 10,11,12 and caused 63 fatalities at Ontake volcano (Japan) in 2014 13,14,15,16. In both cases, no clear warning signals were reported. Both the detection of early warning signals, the quantification of both the hydrothermal volumes involved and the amount of energy available, constitute new challenges to modern volcanology. As quoted by several experts who commented the Ontake eruption, theses challenges deserve the development of new techniques providing new data type and of new concepts in data analysis and information processing^13^,^14^,^16^.

The aim of this paper is to demonstrate the possibility to use muon radiography to monitor density changes in the shallow hydrothermal system inside the lava dome of the La Soufrière of Guadeloupe. As will be shown in the remaining of this paper, we clearly detect density changes related to modifications of the vent activity visible at the volcano’s summit. Quantitative attributes like time-constants, concerned volumes and estimates of the amount of thermal energy available may be derived from muon radiography data.

The paper is organized as follows. First, we recall the main principles of cosmic muon radiography. Then we present the experiment performed on the La Soufrière of Guadeloupe during Summer 2014, at the same time that the North-Napoleon fumarole appeared. In a second stage, we present the data and, after eliminating the possibility of artefacts caused by atmospheric perturbations, we provide evidence of large mass movements within the lava dome. Finally, we briefly discuss the consequences for the dynamics of the shallow hydrothermal system.

## The muon radiography experiment

Muon radiography aims at recovering the density distribution, *ρ*, inside the object of interest (in our case the La Soufrière of Guadeloupe) by measuring its screening effect on the cosmic muon flux^17^,^18^,^19^,^20^. The material property inferred with muon radiography is the opacity, 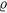 [g.cm^−2^], which measures the amount of matter encountered by the muons along their travel path, *L*, across the rock mass to image,


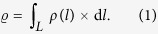


Muons lose energy through matter by ionisation processes at a typical rate of 2.5 MeV per opacity increment of 1 g.cm^−2^. The tomography muons we detect are high energy particles (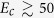) we can approximate to travel along straight trajectories for the spatial resolution we are interested in. The muons incident flux is nearly stationary, azimuthally isotropic and mainly depend on the zenith angle^21^,^22^. Simple flux models can be used to determine the target screening effects^23^,^22^. These properties have been used for 10 years to image volcanoes internal structures^24^,^25^, and more recently to monitor mass variations linked to their activity^26^,^27^.

**Figure 1:**
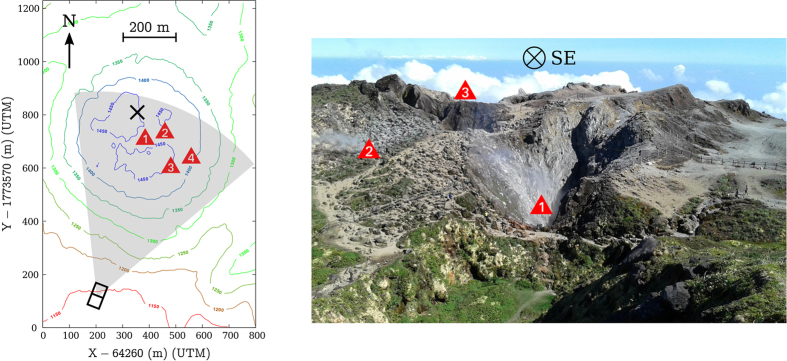
*Left:* La Soufrière of Guadeloupe topographic map. The grey surface delimits the region scanned by the muon telescope represented by the black boxes. The red triangles refer to the different active zones visible at the dome surface (1: Tarissan pit, 2: Napoléon-Nord fume, 3: Cratère Sud, 4: Gouffre Breislack and Gouffre 56). *Right:* The La Soufrière’s different active vents as seen from the black cross on the left picture.

**Figure 2:**
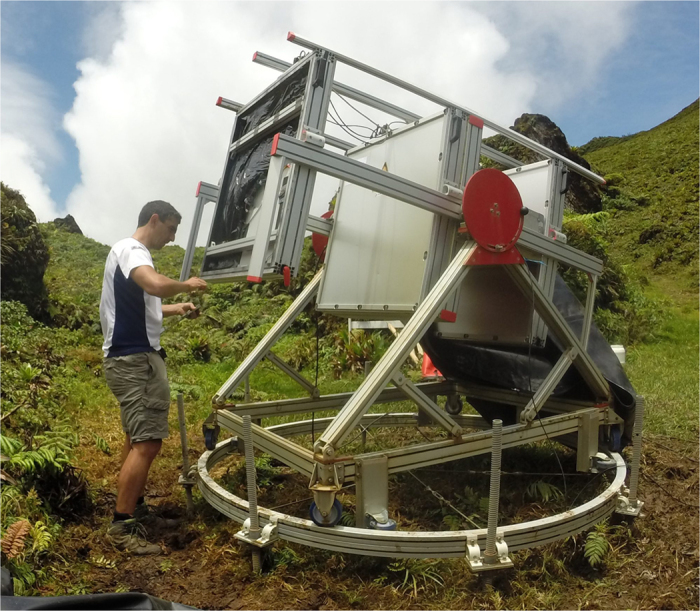
Photography of a muon telescope under maintenance on the La Soufrière of Guadeloupe. The three grey boxes are the three detection matrices. The fourth box, placed just behind the central matrix, contains the telescope steel shielding.

Our cosmic muon telescopes^28^ are equipped with 3 plastic scintillator matrices counting 16 × 16 pixels of 5 × 5 cm^2^ (Fig. 2). We set the distance between the front and rear matrices to 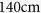 in order to span the entire lava dome from a single point of view (Fig. 1). The combination of pixels defines a total of 31 × 31 lines of sight with a spatial resolution of about 25 m at the lava dome center. A 7cm–thick steel shielding is placed just behind the center matrix in order to filter background low energy particles from the tomography signal^30^,^31^,^32^. The matrices geometrical arrangement fixes the telescope acceptance function 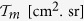 which controls the flux captured by the instrument on each line of sight **r**_*m*_. As shown below, the acceptance has a direct influence on the number of muons counted in a given period of time and, consequently, controls the statistical uncertainty of eventual flux variations caused by density changes in the volcano^22^,^29^.

The present dataset runs from July 7^th^ to August 5^th^ and from August 15^th^ to October 10^th^ 2014. The telescope was placed South-South-West at the lava dome basis (Fig. 1), the axial line of sight was oriented *β*_0_ = 25° Eastward and the zenith angle set to *γ*_0_ = 75° (the telescope is horizontal when *γ*_0_ = 90°).

We standardized the raw data processing was standardized. Data reduction and filtering include : events time coincidence (on the 3 matrices), particle time-of-flight^33^,^34^, alignment of the fired pixels, number of pixels activated in the rear matrix. This number is larger when particles other than muons (electrons and gamma photons) start showering in the steel shielding. We estimate the resulting background flux to be below 0.05 day^−1^.sr^−1^.cm^−2^), which is good enough to precisely make the discussion section volume and mass computations^30^,^31^,^32^ (the article other conclusions can be recovered using only the relative flux time variations). The resulting data set is a sequence 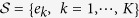 of events *e*_*k*_ attributed to cosmic muons arriving in the telescope front. Each event has a time-stamp *t*_*k*_ and is assigned to one particular line of sight **r**_*m*(*k*)_.

Once obtained, the sequence 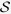 is used to compute the muons number *N*_*m*_ (*t*, Δ*t*) for each line of sight **r**_*m*_ during a time period Δ*t*. It must be corrected from the acceptance function 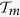 to recover the absolute flux 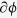 and estimate absolute opacity variations.

An additional processing stage specific to the present study (see Methods section below) consists in merging several adjacent lines of sight in order to improve the signal-to-noise ratio. For lines belonging to a subset *ε*,


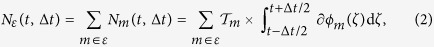


where 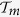 and 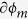 are respectively the acceptance function and the absolute flux in the line of sight **r**_*m*_. Let us precise that more than the telescope geometry, 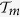 also depends on the detection efficiency and on the numerical algorithms used to filter the proper events. We evaluate 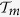 through a calibration experiment made previously to the tomography measurements. In the following we use 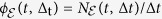 the particle flux at *t*, averaged on a Δ*t* time window. As explained in the Methods section, the lines of sight belonging to a given subset *ε* must share a similar time-variation curve of the muon flux. Such a merging increases the effective acceptance and improves the time resolution. However it induces a decrease in the angular resolution induced by the merging of the small solid angles spanned by the lines of sight.

The telescope mechanical stability is an important issue for monitoring experiments. Changes in the telescope orientation may occur because of the strong mechanical constraints the instrument has to support, especially for measurements under open sky where about 300 kg of steel shielding are necessary as explained above. The ground below the telescope may also slightly move as the Soufrière of Guadeloupe is subject to heavy rains all the year long. Periodic checks of both the orientation and inclination of the telescope frame did not reveal any changes during the experiment. The overall telescope acceptance is monitored permanently on the data themselves (using the responses of open-sky oriented lines). No significant changes have ever been observed. This is also confirmed by the Principal Component Analysis (PCA, see Methods section below) used to merge the lines of sight into coherent domains, since it is able to identify and remove global shifts in all lines of sight. Such an effect was not detected during the processing, we thus conclude that no instrumental bias alter the data.

## Evidence of opacity changes inside the La Soufrière lava dome

**Figure 3:**
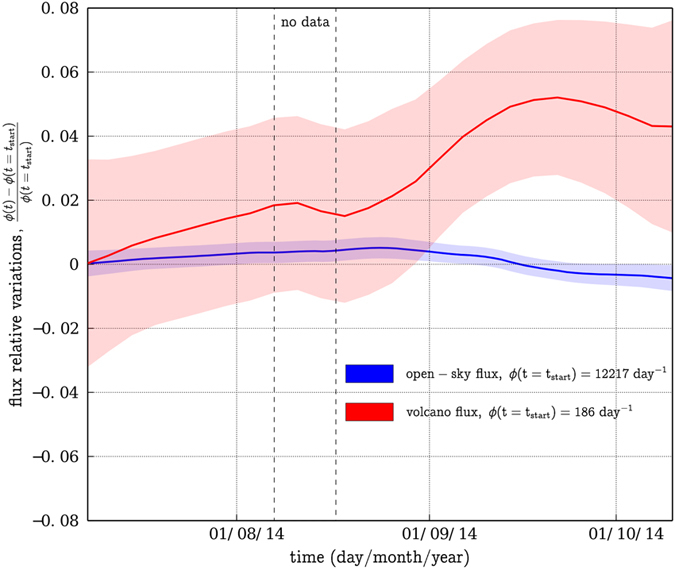
Relative muon flux measured during Summer 2014. In blue: open-sky axes. In red : axes point through the La Soufrière of Guadeloupe volcanic dome. The transparent surfaces associated to each curve delimit the 95% confidence interval. The fluxes are computed using a 30 days large Hamming moving window. The vertical black dotted lines delimit the small period during which the muon telescope was not operational.

The red curve in Fig. 3 shows the muon flux global relative variations through the La Soufrière lava dome. This curve has been obtained by applying eq. 2 to the subset *ε*_dome_ of all lines of sight that pass through the volcano. The blue curve of Fig. 3 corresponds to the subset *ε*_sky_ of the lines of sight directed toward the open-sky above the volcano. The transparent surfaces represent the 95% confidence interval. The flux data were smoothed over a 30 days large Hamming window. This smoothing interpolates the data gap delimited by the two vertical black dotted lines. Figure 3 red curve shows a conspicuous increase of about 5% of the muon flux during the whole observation period. Such an increase is not visible in the open-sky flux which instead slightly decreases by about 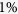 (Fig. 3 blue curve). This indicates that the muon flux increase across the lava dome is likely to be primarily caused by a decrease of the volcano bulk opacity.

**Figure 4:**
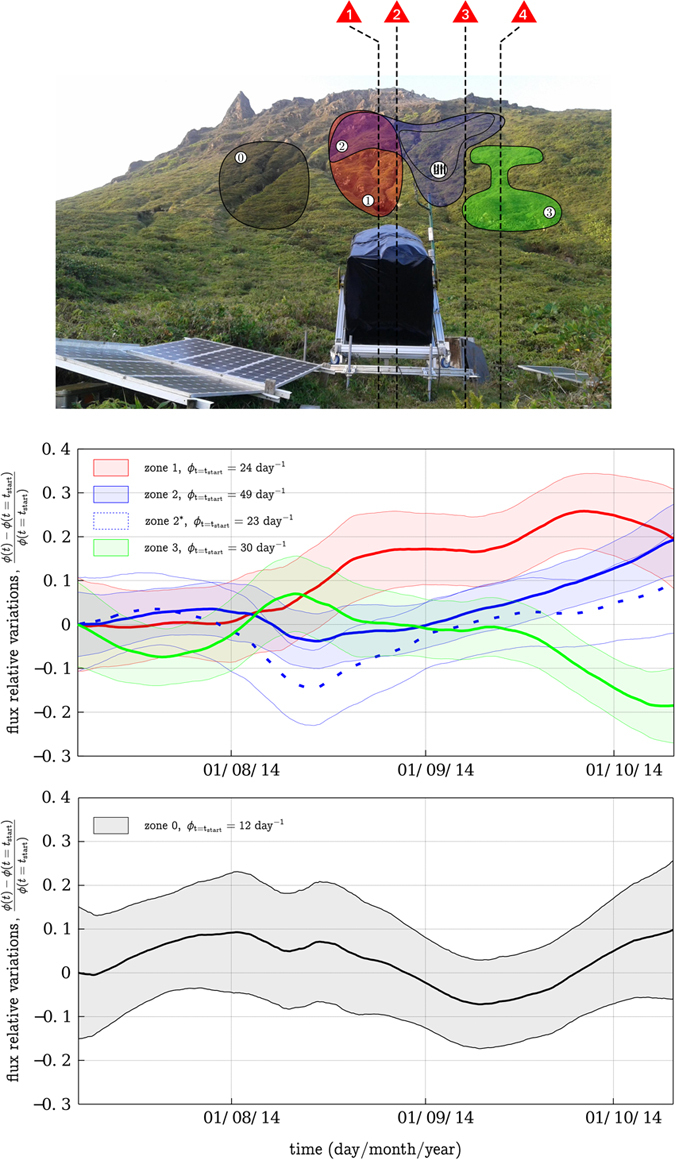
*Top:* The La Soufrière of Guadeloupe as seen from the muon telescope. The coloured regions refer to the regions from which we could extract coherent temporal signals. The red triangles refer to the different active zones visible at the surface of the dome azimuths (Fig. 1). *Middle:* The relative muon flux variations associated to the top picture regions *ε∈* {1, 2, 2*, 3} during Summer 2014. The transparent surfaces associated to each curve delimit their respective uncertainty with a 95% confidence interval. The fluxes are computed using a 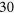 days large Hamming moving window. *Bottom:* Same as the middle graph for the control region *ε*_0_.

In order to identify the locations in the lava dome where the muon flux variations are the most important, we performed a PCA (see Methods section) on all *N*_*m*_(*t*) series. The analysis identifies 3 domains where the muon flux time-variations are similar. Each domains lines of sight were merged to compute 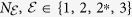. 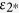 is a *ε*_2_ subset. We chose it so that it does not have any observation axes in common with *ε*_1_ in order to print *ε*_2_ specific variations. Figure 4 shows the 4 identified domains and their associated muon flux time series. We add a fourth region *ε*_0_ where no variations could be extracted by the PCA analysis and that we show as a control zone. We observe relative variations as large as 20%. We exclude from the PCA instrumental or atmospheric effects which would have impacted all the telescope observation axes. The unambiguous spatial localization and the diversity of the different temporal trends appear to be the strongest argument in favour of explaining the variations by the volcanic activity itself. Statistical considerations concerning time-resolution and potential conclusions on the lava dome hydrothermal activity are briefly discussed in the next two sections.

### Atmospheric effects

The cosmic muons are produced in the so-called extended atmospheric showers (EAS) which initiate in the upper atmosphere, at an altitude *z*_*μ*_ ≈ 15–20 km, when primary cosmic ray particles (mainly protons) collide with oxygen and nitrogen atoms. When the thermal state of the atmosphere changes, both the air density and *z*_*μ*_ are slightly modified, producing small variations in the muon flux *ϕ* with respect to its average 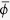. For the present analysis, we use a linear empirical relationship^29^,^35^,^36^,


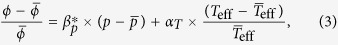


where *p*[hPa] is the ground pressure, 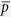 the local average ground pressure, 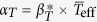 the modulation coefficient and *T*_eff_ the effective temperature. This temperature is an approximation of *T*_*μ*_, the atmosphere temperature at the high energy muons production altitude *z*_*μ*_, not to be misinterpreted with the ground temperature. It corresponds to an estimate of the atmosphere temperature where high-energy muons are produced, at the very beginning of the EAS development.

The coefficients 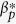 and 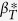 are computed through linear regressions. They mainly depend on the measurement site altitude and latitude^37^, and on the cut-off energy *E*_*c*_(*ρ*) corresponding to the screening caused by the amount *ρ* of matter facing the telescope^38^, a primary importance in applications aiming at imaging large geological structures.

The barometric coefficient 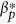 is negative since an increase in *p* induces an increase in the air column opacity. It makes the atmosphere harder to go through for the muons and reduces their total flux. However it mainly affects the soft muons with an energy less than a few GeV. 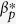 is a decreasing function of *E*_*c*_. This is due to the fact that the differential energy spectrum of cosmic muons is a sharply decreasing function. Consequently, as *E*_*c*_(*ρ*) increases, the number of low-energy muons stopped by an increase of atmosphere opacity is smaller. In the SHADOW experiment^29^ where *E*_*c*_ ≈ 1GeV, we got 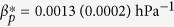. For the present experiment, the opacity ranges from 200 to 2000 mwe and 50 < *E*_*c*_ < 675 GeV. Numerous studies have measured or computed 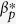 for various cut-off energies^37^,^39^, and in our case we have 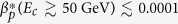. At the summit of the La Soufrière, 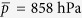 and pressure variations 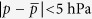. The associated muon flux relative variations are less than 0.1%, which is largely below the muon flux variations reported in Figs 3 and 4.

The second right-hand term of eq. 3 represents the temperature effect, which has already been parametrized multiple times in the literature^36^,^38^,^40^. We adopt here the parametrization of Barrett *et al.*^38^,^40^ who define *T*_eff_ as the atmosphere temperature vertical average weighted by the cosmic pions disintegration probability. From the pions interaction probability with the atmosphere molecules (increasing with density) and their decay probability into muons (also affected by the local density), one expects 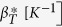 negative for low energy particles (roughly^41^ for *E*_*c*_ below 10 GeV), and positive for high energy particles.

**Figure 5:**
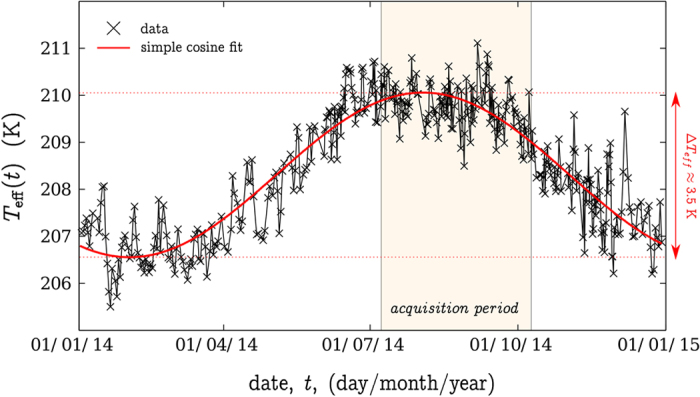
Effective temperature *T*_eff_ estimated from Le Raizet daily launched atmospheric sounding balloons (black crosses). The red curve is a simple fit using a cosine function. The data was extracted from the University of Wyoming website (http://weather.uwyo.edu/upperair/sounding.html).

For the la Soufrière de Guadeloupe experiment, we expect a negative *α*_*T*_ for the open-sky lines of sight, as most of the detected muons have a low energy and a positive *α*_*T*_ for the lines of sight crossing the volcano. Figure 5 shows the *T*_eff_ time series for the whole year 2014 in Guadeloupe using meteorological sounding balloons data. As can be observed, *T*_eff_ is well correlated with the seasons and it variations amplitude is about 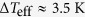. This value is very small because the Guadeloupe archipelago is close to the equator (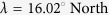) where seasonal effects are almost absent. It should be compared, for example, to the *T*_eff_ annual variation 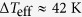 observed at the ICECUBE detector in Antarctica (*λ* = 89.59° South). For the ICECUBE experiment^42^, 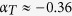 for the open-sky muon flux (*E*_*c*_ ≈ 1 GeV), and 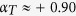 for high-energy muon flux (*E*_*c*_ ≈ 400 GeV).

The present dataset being quite short, the sampling of *T*_eff_ annual variation is affected by large uncertainty: *α*_*T*_ = +1.05 (1.45) (95% confidence interval) for the open-sky flux (Fig. 3 blue curve). Using the *α*_*T*_ values computed as a function of *E*_*c*_ through numerical models and measured with various underground experiments^41^,^42^,^43^,^44^ (e.g. Fig. 7 from Adamson *et al.*^41^), we obtain 0.2 < *α*_*T*_ < 0.8 for the lava dome opacity range 200 mwe < 2000 mwe. With these values, the expected relative muon flux variations due to temperature effects should not exceed 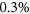 during the acquisition period, which is more than one order of magnitude under the detected fluctuations (Fig. 3 red curve and Fig. 4).

From the results discussed in this section, we conclude that neither the pressure effects nor the temperature effects may explain a significant part of the muon flux variations observed through the lava dome.

### Statistical time resolution

The temporal fluctuations one can extract from the muon flux signal are intrinsically limited by the statistical noise. We previously demonstrated^29^ that the minimum acquisition time 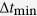 necessary to extract a relative variation *ε* from the average detected flux 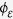 reads,


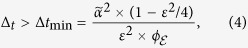


with 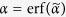 the confidence interval chosen to validate the statistical hypothesis.

A single observation axis that crosses the la Soufrière of Guadeloupe in this experiment detects between 1 and 5 particles per day. Then according to eq. 4 it can at best extract during our 95 days experiment a respectively 40 and 20% relative flux variation (with a 2*σ* precision). We could not detect such strong fluctuations in la Soufrière of Guadeloupe on this time-scale on a single axis. The solution is then to sum the signals from the different observation axes (see Methods section). Doing so we increase 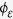, and improve both the time and amplitude resolutions of the fluctuations (respectively 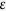 and 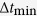). As a counterpart, we deteriorate their spatial localization.

As presented on Fig. 4, we are finally able to extract *ε* ≈ 10% relative fluctuations on a 30 days time-scale in regions regrouping from 21 (with zone *ε*_1_) to 42 axes (with zone *ε*_2_). Taking an average flux of 3 particles per day per observation axis, we respectively get with eq. 4 minimum acquisition times ranging from 13 to 24 days. These numbers are coherently below the 30 days Hamming window mentioned previously.

## Discussion and Conclusions

The data presented and discussed in the preceding sections show that huge opacity variations occurred in the La Soufrière lava dome during Summer 2014. Using spatial PCA, theses variations revealed that the observed opacity changes are organized as separate regions located in the volcano southern half underneath the active fumarolic areas visible on the lava dome summit (Fig. 4). Both domains *ε*_1_ and *ε*_2_ show a conspicuous increase of their muon flux roughly starting August 1^st^ for *ε*_1_ and August 10^th^ for *ε*_2_. In the same period, a decrease of the muon flux through domain *ε*_3_ starts August 8^th^. The only plausible explanation is that these rather fast mass changes originate from fluid movements inside the dome^45^.

**Figure 6:**
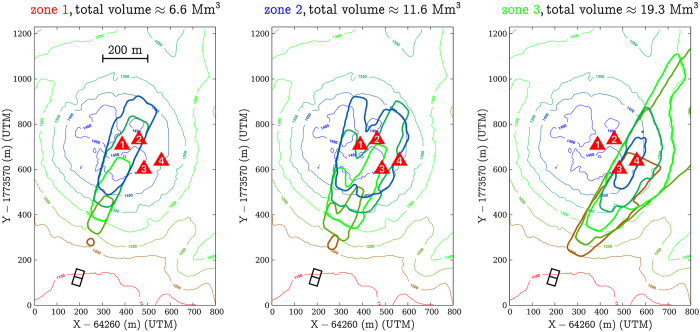
Plan view of the volume scanned by the muon telescope for the three domains *ε*_1_, *ε*_2_ and *ε*_3_ represented on Fig. 4. The joined squares located SouthWest represent the telescope, and the red triangles refer to the active areas visible on the lava dome summit plateau (Fig. 1).

The muon flux variations, i.e. opacity fluctuations, in each domain may be used to estimate their associated mass changes. However, since the lines of sight are small conical volumes with their apex located on the telescope, the mass changes depend on the location of the concerned volume along the line of sight. We draw the entire volumes intercepted by the telescope for the 3 considered lines of sight subsets on Fig. 6. Let us assume that an opacity change Δ
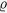
_*m*_ is observed for a given line of sight **r**_*m*_ and that a density variation 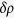 occurs somewhere in a volume *V*_*m*_ along this line. The segment length *L*_*m*_ of 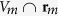 reads (see eq. 1),


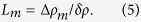


If *V*_*m*_ is located at a distance *L*_0_ from the telescope,


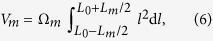


where 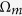 is the small solid angle spanned by the line of sight **r**_*m*_. The total volume *V*_*ε*_ is obtained by summing the volumes of all lines of sight belonging to a given domain *ε*,


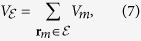


and the mass change associated with *V*_*ε*_ is given by,


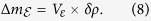


**Figure 7:**
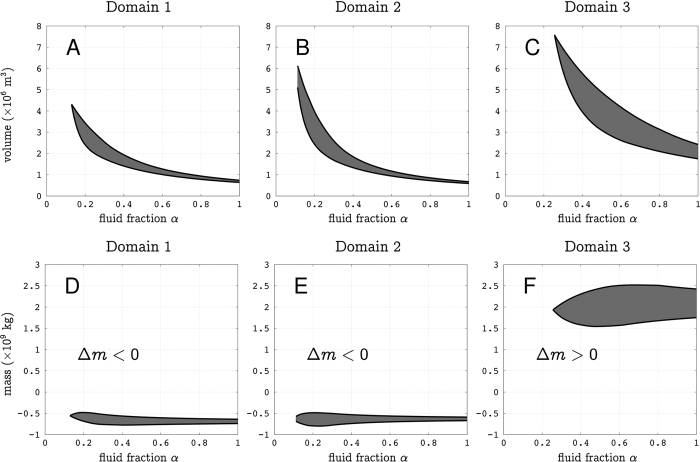
*Top*: Volume ranges *V*_ε_ for the domains *ε*_1_ (**A**), *ε*_2_ (**B**) and *ε*_3_ (**C**) as a function of the fluid fraction *α. Bottom:* Ranges of mass change Δ*m*_*ε*_ for the domains *ε*_1_ (**D**), *ε*_2_ (**E**) and *ε*_3_ (**F**) as a function of the fluid fraction *α*.

To compute the volumes *V*_*ε*_ and their corresponding mass changes 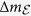, we need to know both 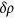 and *L*_0_. The density change is assumed to be caused by fluid movements where liquid is replaced by gas (air or steam) or inversely. In such a case, 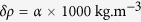, where *α* is the volume fraction occupied by the fluids in the rock matrix. The choice of *L*_0_ may be guided by geological information about the locations where fluid movements are likely to occur inside the lava dome. In the present study, we consider that fluid movements occur under the active areas that occupy the Southern-East quarter of the summit plateau^45^ (see the red triangles on Fig. 1). The volumes and mass changes corresponding to *ε*_1_, *ε*_2_ and *ε*_3_ are shown in Fig. 7.

**Figure 8:**
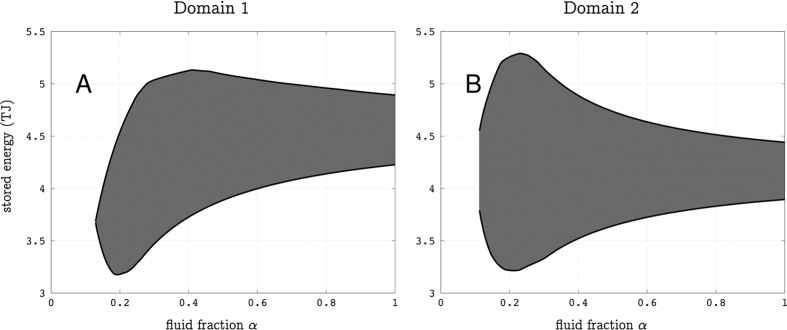
Energy brought by steam in domains *ε*_1_ (**A**) and *ε*_2_ (**B**) as a function of the fluid fraction *α*. Values computed for an absolute pressure p = 6 bars.

It may be expected that the positive mass variation of *ε*_3_ (Fig. 7F) is due to the filling of a perched aquifer with rain water. Taking a surface *S* = 1.5 × 10^5^ m^2^ for the summit plateau of the lava dome and a total amount of rain of 2 m during August and September, we obtain an upper bound of *S* = 3 × 10^8^ kg of water input on top the lava dome. However, most of this water is rapidly evacuated through run-off at the surface and it is very unlikely that filling *ε*_3_ with rain water is the cause of the positive mass variation Δ*m*_3_. A more reasonable hypothesis is that this mass movement is due to fluid flow from *ε*_1_ and *ε*_2_ into *ε*_3_. This is sustained by the fact that the positive mass variation Δ*m*_3_ is anti-correlated with the negative time-variations of mass associated with *ε*_1_ and *ε*_2_ (Fig. 4). Actually, the mass-variation ranges (Fig. 7) allow to have an almost vanishing mass budget, with the mass increase in *ε*_3_ compensating the decreases in *ε*_1_ and *ε*_2_. In such a case, we expect that high-pressure steam formed in *ε*_1_ and *ε*_2_ and pushed liquid into *ε*_3_. Considering that the steam pressure must be sufficient to push liquid and assuming a maximum reservoir depth of 50 m, we take an absolute pressure *p* = 6 bars. This gives a thermal energy storage of about 4.5 × 10^12^ J in each reservoir *ε*_1_ and *ε*_2_ (Fig. 8).

**Figure 9:**
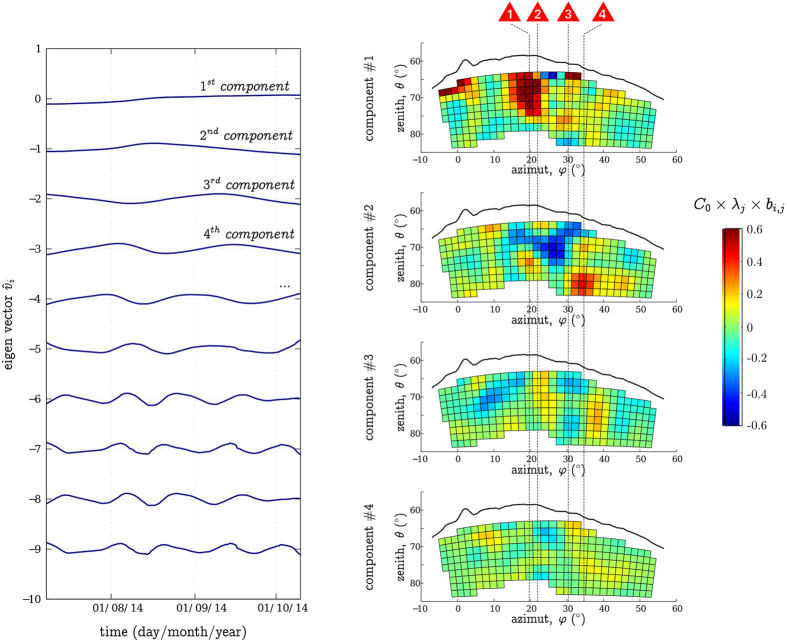
*Left*: The first ten eigenvectors 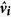 extracted from the Summer 2014 muon tomography data. All the eigenvectors should be centred but here we added an increasing offset so that the trends do not superimpose on each other. To construct them each observation axis was previously smoothed using a 30 days large Hamming moving window. *Right:* Graphical representation of the four first eigenvector contributions to the measured signal. Each box corresponds to an independent observation axis *i* and the associated color relates to 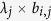 (*j* going from 1 to 4). *C*_0_ is a scaling constant. The black solid curve delimits the interface between the volcanic dome and the sky. The red triangles refer to the different active zones visible at the surface of the dome azimuths (Fig. 1).

To conclude, PCA appears to be a fitted solution to regroup the telescope observation axes. We could extract 3 clear domains associated with various temporal signals that are probably linked to the different physical processes occurring into the volcano. PCA being a linear decomposition, it permits to consider an alignment of different zones on a single observation axis which is an intrinsic problem to tomography imaging from a single point of view. It is the case here with the zones *ε*_1_ and *ε*_2_ (Figs 4 and 9). The different domains geometrical shapes, as well as their alignment with the surface active vents are probably the best argument in favour of the detection of a volcanic signal because no prior information was injected into the analysis. *ε*_2_ even shows a clear connection between two active regions.

However let us mention four factors that limit this approach. First our analysis assumes the temporal trends occur in fixed areas while we can expect the regions to change size, to merge in between each others, or to divide into smaller independent ones. Second the PCA algorithms searches the simplest possible solution. Many other combinations can be imagined, and for example two regions that completely superimpose onto the other as seen from the telescope cannot be distinguished (Fig. 4). Third PCA zoning may be biased by the telescope observation axes acceptance variations. The central axes have a higher sensitivity than the border ones^29^,^43^. This probably explains why we could not extract any fluctuations on the telescope scanning window borders (Fig. 9): the associated axes have a weaker flux and thus a smaller weight in the PCA decomposition. Finally one could fear that when processed on such short data sets, PCA would tend to regroup observation axes with similar statistical noise. We believe it is not the case here as the regions presented on Fig. 4 are spatially coherent and aligned with the La Soufrière active vents, and because we detect fluctuations with a precision up to 4*σ* (see Fig. 4).

More constrains could be brought to the dynamics of the shallow hydrothermal system of the La Soufrière of Guadeloupe’s lava dome with additionnal cosmic muon telescopes deployed around the volcano to provide radiographies taken under different angles of view, and also using recent 3D structural imaging of the volcanic dome^45^. This will allow to perform 3D reconstruction of the volumes *ε*_1_, *ε*_2_ and *ε*_3_ and reduce the ranges of mass changes in Fig. 7. An even better reconstruction could be reached by merging the muon radiography measurements with continuous gravimetry data on the dome summit^46^. We estimated that the mass changes discussed in this study would generate gravity anomalies around a hundred microgal, which would be easy to measure with a standard gravimeter.

To conclude, we demonstrated that muon radiography provides unprecedented knowledge about the La Soufrière of Guadeloupe shallow hydrothermal system dynamics. We hope that it will become a standard geophysical monitoring technique to both improve our understanding of the physical processes at work and bring useful informations to assess hazard level. For example it could become a efficient proxy to localize pressurized reservoirs and evaluate their mechanical stability in order to prevent potential volcanic blasts.

## Methods – Pixel merging through principal components analysis

As explained previously, a telescope single observation axis does not collect enough particles to statistically distinguish variations in the muon flux linked to the volcanic activity on a monthly time-scale. We can solve the problem regrouping various observation axes in order to increase the signal intensity. Thus, we come up with the following two-folds problematic: how to regroup the observation axes in order to efficiently extract temporal trends without introducing any prior information that may bias the solution ?

We propose here a solution using Principal Components Analysis (PCA, also called the Karhunen & Loeve Transformation^47^,^48^). Let us note 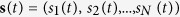, where **s**(*t*) is a vector containing the Summer 2014 temporal signals 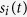 associated to the different observation axes that cross the volcano. PCA gives an optimal linear decomposition of the 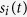 on an orthonormal basis 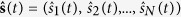 calculated iteratively from the signal itself,

- **Iteration 1:** we search 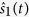 such as,

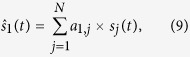


where the coefficients *a*_1,j_ are estimated by minimizing the quadratic norm *ε*_1_,


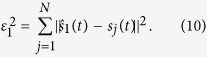

- **Iteration i :** we search 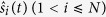 such as,

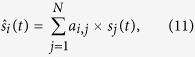


where the coefficients 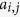 are estimated by minimizing the quadratic norm 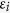,


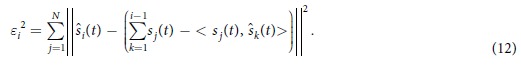


The resulting basis is orthogonal, we normalize it,


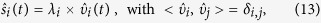


where 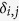 is the Kronecker Delta. 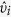 is the 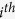 eigenvector and 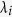 its associated eigenvalue.

Finally, usinq eqs 11 and 13 we get the decomposition,


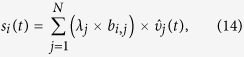


where 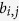 are the square matrix 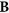 coefficients, with 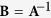, 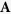 being the *N* × *N* square matrix associated to the 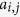 coefficients.

Let us recall that PCA, as shown in eq. 12, assumes the signals 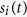 are affected by a Gaussian noise. This condition is not fulfilled for all the telescope observation axes going through the volcano, and for the time-scale we are interested in, because of the muon flux weak intensity. In order to overcome this problem, we merge observation axes into groups of four neighbours and the flux is computed with a 30 days large Hamming moving window.

By construction, the eigenvectors are less and less representative of the input data (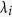 is decreasing with i). Usually the first ones represent the global trends that are redundant on the different input signals while the last ones permit to reconstruct the little and specific discontinuities (mainly the noise). The eigenvectors are interesting four our study, because there is a possibility that they characterize different physical processes occurring inside the volcano, but we can also use the coefficients 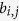 to map their respective contribution in the different regions of the dome.

Figure 9 shows the first ten eigenvectors (on the left) and, for the first four, their associated contribution on the different observation axes as seen from the telescope (on the right). Only the two first eigenvectors show fluctuations occurring on time-scales larger than a month, which can statistically be recovered considering the muon flux intensity (see the statistical time resolution section). The next eigenvectors present quicker fluctuations that characterize the statistical noise. Very clear large regions appear on the two first contribution maps (associated with the two first eigenvectors). The first component reveals a large coherent domain *ε*_1_ aligned with the Tarissan pit (TP) and the North-Napoléon fume (NN). The second component shows two independent and anti-correlated regions: a V-shape zone 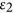 which left arm is aligned with TP and NN, and right arm with the Cratère Sud, the Gouffre Breislack (GB) and the Gouffre 56 (G56), and a bone-like zone 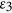 aligned with GB and G56. The next maps do not present any clear zone, the coefficients are small and appear to be randomly distributed in between the different observation axes (the little spatial coherence that appears is due to the neighbours merging mentioned previously).

## Additional Information

**How to cite this article**: Jourde, K. *et al.* Muon dynamic radiography of density changes induced by hydrothermal activity at the La Soufrière of Guadeloupe volcano. *Sci. Rep.* **6**, 33406; doi: 10.1038/srep33406 (2016).
